# The Immune, Inflammatory and Hematological Response in COVID-19 Patients, According to the Severity of the Disease

**DOI:** 10.3390/microorganisms11020319

**Published:** 2023-01-27

**Authors:** Felicia Trofin, Eduard-Vasile Nastase, Andrei Vâță, Luminița Smaranda Iancu, Cătălina Luncă, Elena Roxana Buzilă, Mădălina Alexandra Vlad, Olivia Simona Dorneanu

**Affiliations:** 1 Microbiology Department, University of Medicine and Pharmacy “Grigore T. Popa”, 700115 Iasi, Romania; 2 Clinical Hospital of Infectious Diseases “Sf. Parascheva”, 700116 Iasi, Romania; 3 Infectious Diseases Department, University of Medicine and Pharmacy “Grigore T. Popa”, 700115 Iasi, Romania; 4 National Institute of Public Health, Iasi Regional Center for Public Health, 700465 Iasi, Romania; 5 “Sf. Maria” Children Emergency Hospital, 700309 Iasi, Romania

**Keywords:** immune response, IL-6, SARS-CoV-2, COVID-19, severity forms

## Abstract

Introduction: The aim of this study was to evaluate the immune and inflammatory responses in COVID-19 patients by dosing specific IgM and IgG total antibodies and interleukin 6, correlating them with the hematological and biochemical blood parameters and comparing them by the form of the disease. Materials and methods: One hundred twenty-five patients with polymerase chain reaction-confirmed COVID-19, hospitalized between 15.03.2020 and 1.07.2020 in the Clinical Hospital of Infectious Diseases “Sf. Parascheva” Iaşi, were tested by chemiluminescence for the presence of anti-SARS-CoV-2 IgM and IgG and IL-6 in the serum. The results were correlated with the results of the CBC count and serum biochemical parameters detected on the admission day. The patients presented different forms of the disease (asymptomatic, mild, moderate, severe, and critical) according to World Health Organization (WHO) criteria for the clinical management of COVID-19. Results: The amplitude of the immune response was directly correlated with the form of the disease. In the asymptomatic/mild form patients, the IL-6 and CRP concentrations were significantly higher and eosinophil count was significantly lower compared with the reference interval. In the moderate form, the concentrations of IL-6, CRP, and IgG were significantly higher, compared with the reference interval, while eosinophil count and eGFR were significantly lower. In severe/critical COVID-19 patients, IL-6, CRP, NLR, PLR, glucose, AST, urea, creatinine, and eGFR were significantly higher compared with the reference interval, while eosinophil count was significantly lower. IL-6 boosted in all forms of COVID-19, with a major increase in severe and critical patients. IL-6, neutrophil count, % neutrophils, NLR, PLR, CRP, AST, and urea increased with the severity of the SARS-CoV-2 infection, and the lymphocyte count, % lymphocytes, eosinophil count, % eosinophils, and hemoglobin decreased with the increased severity of COVID-19. Conclusions: The amplitude and the moment of appearance of the immune response depended on the form of the disease. IgM generally occurred in the first 14 days of illness, and IgG appeared beginning with the second week of disease. IgG titer increased rapidly until the fourth week of disease and decreased slowly after 4 weeks. The amplitudes of all the tested inflammatory and serological markers depended on the COVID-19 form, increasing somewhat in the moderate forms and even more in the critical ones. The lymphocyte and eosinophil count are able to predict the risk of severe COVID-19.

## 1. Introduction

The importance of coronaviruses (CoVs) was emphasized by doctors and researchers in 2002, with the outbreak of the severe acute respiratory syndrome (SARS) virus. Until then, CoVs had not generally been considered important pathogens for humans, as circulating strains caused only mild infections even in immunocompromised hosts [1]. In December 2019, a new virus, identified as the severe acute respiratory syndrome virus-2 (SARS-CoV-2), reminded us that CoVs are a severe threat to global health [2].

Laboratory blood tests are recommended to monitor the evolution and the prognosis of the coronavirus disease 2019 (COVID-19) [3]. Antibody detection plays an important role in the epidemiology of the infection because they can reveal the populations that had an immune response after vaccination or natural infection [1,4].

Anti-SARS-CoV-2 immunity is given by the sum of cell-mediated immune response and specific antibodies production. Identifiable antibodies in COVID-19 are immunoglobulins (Ig) M, G, and A. IgM occurs in the serum of patients approximately 5–7 days after the onset of the disease, while IgG occurs 10–21 days after the onset and may persist for a long time [1,2,4,5]. Many authors argued that the immune response in SARS-CoV-2 infection targets the receptor-binding domain (RBD) or other parts of the protein subunits [1]. Some authors described an early humoral immune response of IgA in COVID-19 patients, which provides a more rapid diagnosis and better tool for prognosis compared with IgM [6,7,8,9]. The medium seroconversion time for IgA is 4–6 days after the onset of symptoms [6].

The IL-6 concentration is known to predict the development of the severe COVID-19 and of the hypoxemia that will need hospitalization [10,11,12]. The severe form of this infection is also associated with a higher serum concentration of C-reactive protein (CRP) [11], glucose, alanine aminotransferase (ALT), aspartate aminotransferase (AST), creatinine [10,13,14], urea [14], estimated glomerular filtration rate (eGFR) [14], neutrophilia, and low lymphocytes and eosinophils count, respectively [10,13,15], and higher amount of serum ferritin [16]. 

A neutrophil-to-lymphocyte ratio (NLR) of 2.90 and a platelets-to-lymphocyte ratio (PLR) of 186 have good specificities for the association with severe forms of COVID-19. Reported cut-off values were 3.3 and 180 for NLR and PLR, respectively [15].

There are four different forms of COVID-19, according to the World Health Organization, and they depend on the severity of the disease: mild, moderate, severe, and critical [17]. Additionally, some infected patients did not develop any symptoms at all, having asymptomatic forms of COVID-19 [18].

Many questions revolve around COVID-19′s serological response. It is currently unclear what the antibodies’ titer requires for immune protection. The lifespan of the antibodies is uncertain. There have also been reported infections with no antibody detection, at least not during the study period [19]. Secondly, it is known that all forms of disease, including the mild ones, may be followed by complications, especially the development of the post-COVID-19 syndrome [20]. Some COVID-19 symptoms may persist for up to 3 months in 10% of patients [18]. Usually, they manifest as one to three of the following symptoms: dyspnea, sleep disturbance, fatigue [21], asthenia, cough, or anosmia [22,23]. To the best of our knowledge, a limited number of studies have correlated the serological parameters with the forms of the SARS-CoV-2 infection, but only few brought up the immune and inflammatory responses or the blood parameters of the asymptomatic or mildly infected SARS-CoV-2 patients. 

The study was motivated by the fact that patients with mild or asymptomatic COVID-19 forms were not studied in detail, and often were even omitted entirely. The mild-form patients are thought to spread the disease more frequently, as they do not seek medical attention or self-implement social restrictions. Therefore, we proposed that the tested parameters should be related to the form of the infection. 

The aim of our study was to quantify the total anti-SARS-CoV-2 IgM and IgG and IL-6 in human serum from COVID-19 patients and to assess if any of the routine blood tests performed on the first day of hospitalization can predict the extent of the inflammatory response or that of the specific antibody production.

## 2. Material and Methods

### 2.1. Study Design and Participants

We conducted a prospective study that analyzed the antibody titers and IL-6 concentration in SARS-CoV-2-infected patients’ serum samples by the form of the disease. 

The inclusion criteria were as follows: a positive SARS-CoV-2 RT-PCR test at admission to hospital, aged over 18 years, and the agreement of the patient to participate in the study.

The exclusion criteria were as follows: treatment with tocilizumab at the time of or in the days before the collection of the serum sample, insufficient amount of serum sample, incomplete hematological or biochemical data from the blood tests at hospital admission or at patient progression, or the patient not agreeing to participate in the study.

We included 125 consecutive patients admitted to the Clinical Hospital of Infectious Diseases “Sf. Parascheva” Iași between 15 March and 1 July, 2020. SARS-CoV-2-positive patients were confirmed by positive RT-PCR through both nasopharyngeal and oropharyngeal swab samples. Depending on the signs and symptoms at the admission, they were diagnosed with different forms of COVID-19 (asymptomatic, mild, moderate, severe, and critical). The total specific IgM and/or IgG antibodies were tested for all patients, but, for economic reasons, we were able to dose IL-6 concentration for only 80 of the included patients. Demographic data (age, sex) and comorbidities were collected for all patients. These data are available in Table S1 in the Supplementary Materials.

The results were corroborated with the form of the disease; CBC, NLR, PLR, CRP, ALT, AST, glucose, urea, creatinine, and eGFR on the day of hospital admission; and the length of hospital stay (LOS).

### 2.2. Collection of Samples

Depending on LOS, 1, 2, or 3 sera from patients were collected. The number of serum samples collected from each patient was correlated with the number of days of hospitalization—one serum sample for patients who underwent 7–12 hospitalization days, two serum samples for patients with 12–20 hospitalization days, and three serum samples for patients with more than 21 hospitalization days—so that we could observe any significant dynamics. The first serum sample was used for anti-SARS-CoV-2 IgM and/or anti-SARS-CoV-2 IgG and IL-6 detection; the following two sera were tested only for specific IgG. The samples were transferred in Eppendorf tubes, frozen at −20 °C, and stored until analyzed.

**Figure 1:**
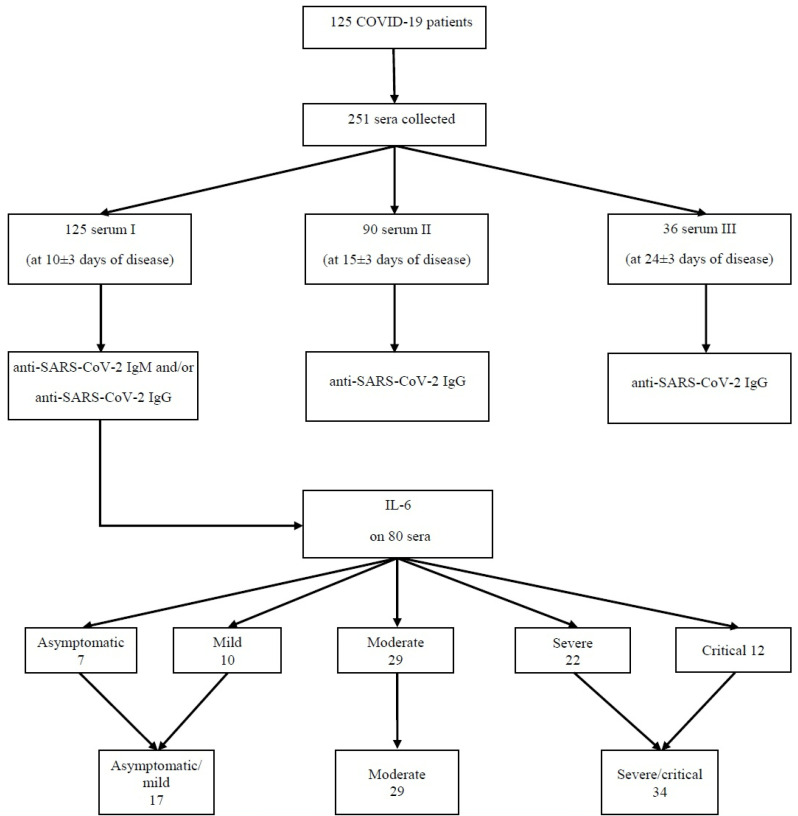
Study flowchart.

A total of 251 sera were collected as follows: for all 125 patients, one sample was taken 10 +/− 3 days from the onset of symptoms; for 90 patients, a second one was taken at 15 +/− 3 days; and for 36 patients from the patients who collected the second sample, a third sample was collected at 24 +/− 3 days after the onset of disease. Fifty-four patients had two serum samples collected and thirty-six patients had three serum samples taken. The sera were sampled from 125 consecutive patients who met the inclusion criteria (Figure 1). 

### 2.3. Analysis of Samples

Luminescence for total anti-SARS-CoV-2 specific antibodies (IgM and IgG, respectively) was detected by a chemiluminescence immunoassay. The light signal, which is measured by a photomultiplier as relative light units, is proportional to the concentration of anti-SARS-CoV-2 IgM or IgG present in the sample. Serum samples were evaluated using the MAGLUMI 2019-nCov IgM (Catalog number: 130219016M) and 2019-nCov IgG (Catalog number: 130219015M) kits (Snibe Diagnostic, Shenzhen, China). The cut off value according to the manufacturer is 1.00 AU/mL for both SARS-CoV-2 immunoglobulins. The manufacturer reported a sensitivity of 78.65% for IgM detection and 91.21% for IgG detection, while the specificities of IgM and IgG were 97.50 and 97.3%, respectively. The results were also analyzed individually as the mean of the concentrations obtained from all patients investigated in each 7-day interval from the onset of the infection.

The IL-6 concentration was quantified by the same method, using the MAGLUMI IL-6 kits (Snibe Diagnostic, Shenzhen, China, Catalog number: 130616004M). The assay is linear between 1.5 pg/mL and 5000 pg/mL.

### 2.4. Ethical Principles

The study complied with the ethical principles stated by the World Medical Association’s Declaration of Helsinki, regarding medical research involving human subjects. The study was approved by the Commission of Ethics of Research from the University of Medicine and Pharmacy “Grigore T. Popa” Iasi, Romania (IRB number: 99), and by the Hospital Ethics Committee (IRB number: 30).

### 2.5. Statistical Analysis

We used the IBM SPSS statistical software version 20 for data analysis. The distribution of the variables was verified using the Kolmogorov–Smirnov test. For the normally distributed variables, we used the Pearson correlation test, and for the others we used the Spearman correlation test. For group statistics, we compared the groups using a one-way ANOVA test. The patients’ blood test results were compared with the reference interval of each parameter using a one-sample *t*-test. We considered a *p* < 0.05 as significant, due to the less than 5% chance probability of the event occurring. The level of significance that we used refers to the α-significance, which measures the probability of false positives. The ROC curve and the area under curve (AUC) values were generated in order to assess the sensitivity and specificity of the tested biomarkers in predicting the risk of a severe form of COVID-19. The conclusions of the studies were supported by the results of the statistics tests. The mean concentration, median, standard deviation, variance, and range of concentrations were calculated using the same software.

## 3. Results

The serological study was conducted on 125 patients. Among these patients 80 were tested for IL-6: seven (8.75%) asymptomatic, ten (12.5%) mild, twenty-nine (36.25%) moderate, twenty-two (27.5%) severe, and twelve (15%) critical patients. They were merged into three groups: 17 (21.25%) patients were placed in the asymptomatic/mild group, 29 (36.25%) patients in the moderate group, and 34 (42.5%) patients in the severe/critical group. This step aided us in the statistical analysis through batch balancing and more significant group sizes (Figure 1). 

The patients were aged between 19 and 88 years old. The mean age was 52 years old for the asymptomatic/mild form, 53 years old for the moderate form, and 59 for the severe/critical form of disease. Forty-five (56.25%) of the patients were female.

In most patients with the asymptomatic/mild form of the disease, IgM became detectable after the 7th day of illness. IgG was found in most patients with asymptomatic/mild forms, beginning with the second week of illness, with the highest concentration recorded in the fourth week of disease. 

In patients with moderate forms, the immune response began in the first week for IgM and in the second for IgG. The maximum value of the concentration mean per week was reached in the first week of hospitalization for IgM and in the fourth week for IgG. 

Almost all patients with severe/critical forms developed both types of antibodies after the seventh day of illness. The maximum mean concentration per week for IgM was recorded in the second week of illness, while for IgG it was recorded in the third week of the disease. 

IL-6 was increased in 74% patients with asymptomatic/mild disease; the mean value for these patients was 19.57 pg/mL. The mean concentration of anti-SARS-CoV-2 IgG in these patients was 25.82 AU/mL. All patients from the other two groups had higher IL-6 levels. In patients with moderate forms, the mean IL-6 value was 73.26 pg mL and the mean anti-SARS-CoV-2 IgG concentration was 36.81 AU/mL. In severe/critical patients the mean values were 149.36 pg/mL and 64.17 AU/mL for IL-6 and anti-SARS-CoV-2 IgG, respectively. 

The mean values of certain blood parameters increased accordingly with the severity of the infection: leucocyte count, neutrophil count, % neutrophils, NLR, PLR, CRP, urea, and creatinine. On the other hand, there are some means that decrease depending on COVID-19 severity: lymphocyte count, % lymphocytes, eosinophil count, % eosinophils, hemoglobin, and eGFR (Table 1). The relevant statistical parameters (mean, σ—standard deviation, median, and inter-quartile range, and variance values) for all the investigated parameters are listed in Table 1 and Table 2.

We used the Kolmogorov–Smirnov test to assess the distribution of the variables. For normally distributed parameters we used the Pearson test to uncover whether there was any correlation between them. The LOS days did not correlate with % neutrophils, lymphocyte count, % lymphocytes, hemoglobin, or platelet count nor with the PLR.

To correlate all the other variables which were not normally distributed (*p* < 0.05), we used the Spearman test. Its results underlined a statistically significant correlation between COVID-19 severity and the IL-6, IgG, neutrophil count, % neutrophils, NLR, PLR, CRP, AST, and urea (Table 3). The lymphocyte count, % lymphocytes, eosinophil count, % eosinophils, and hemoglobin were inversely correlated with the form of the disease (Table 3). LOS correlated significantly only with creatinine (*r* = 0.316, *p* = 0.007) and eGFR (*r* = −0.342, *p* = 0.003).

The IL-6 concentration correlated with the IgG titer, % neutrophils, NLR, PLR, and CRP and negatively correlated with lymphocyte count, % lymphocytes, hemoglobin, and eGFR (Table 4).

The IgG titer was correlated with the leucocyte count, neutrophil count, % neutrophils, NLR, CRP, and ALT but only weakly correlated with the IL-6, PLR, and AST (Table 5). There was a moderate negative correlation between the IgG concentration and the % lymphocytes (Table 5).

Using the One-Sample *t*-Test, we compared the obtained results of the blood tests of our patients with the reference intervals (Table 6). In asymptomatic/mild forms, biomarkers IL-6, CRP, and the IgG were significantly increased (Table 6). Asymptomatic/mild patients displayed significantly lower eosinophil counts and % eosinophils (Table 6). The moderate form of COVID-19 registered significantly higher values of IL-6, CRP, and IgG (Table 6). Patients with moderate forms displayed significantly decreased eosinophil counts and a significantly lower eGFR (Table 6). IgG, IL-6, CRP, NLR, PLR, glucose, AST, urea, creatinine, and eGFR were significantly higher in severe/critical COVID-19 patients. In the same group, eosinophil counts were significantly lower (Table 6).

We compared the blood tests results as dependent variables with the sex of the patient as the independent variable using the independent samples *t*-test. This showed a significantly higher IgG secretion (*p* < 0.001) and NLR (*p* = 0.038) in adult males compared with adult females.

We also compared all the blood parameters according to the COVID-19 severity groups. In this case, we used the one-way ANOVA test (Table 7). The form of the disease was considered as an independent variable and all the other parameters as dependent variables. This test highlighted significant differences between the three groups of COVID-19 severity in IL-6 concentrations, CRP, % neutrophils, lymphocyte count, % lymphocytes, eosinophil count, % eosinophils, NLR, PLR, and urea (Table 7). On the other hand, in IgG levels, leucocyte count, neutrophils, hemoglobin, platelet count, LOS, glucose, ALT, AST, creatinine, and eGFR, the registered *p* was > 0.05.

To appraise the sensitivity and specificity of the investigated biomarkers in predicting the severe forms of COVID-19, we performed ROC analysis. The variables that best predicted the risk of severe COVID-19 were the lymphocyte count (AUC = 0.779), lymphocyte percentage (AUC = 0.752), eosinophil count (AUC = 0.767), and eosinophil percentage (AUC = 0.768) (Supplementary Materials, Figure S1). The corresponding cut-off values were 1.275 × 10^3^/µL (sensitivity = 0.733; specificity =_0.339) for lymphocyte count, 27.9% (sensitivity = 0.733; specificity = 0.288) for lymphocyte percentage, 0.025 × 10^3^/µL (sensitivity = 0.733; specificity =_0.322) for eosinophil count, and 1.05% for eosinophil percentage (sensitivity = 0.533; specificity =_0.153).

The IL-6 concentration and the severe form of COVID-19 are correlated with the presence of comorbidities (Table 8). Forty-six (58.75%) patients had at least one cardiovascular disorder; thirty-two (40%) had associated cardiovascular, metabolic, renal, or endocrinological conditions; and twenty-two (27.5%) had no comorbidities (Table S1, Supplementary Materials).

## 4. Discussion

Until now, most COVID-19 research has been focused on risk factors, such as age [24,25], body mass index [26], sex [27], specific geographical regions [28,29,30], pregnancy [31], ethnicity [32], or specific comorbidities [33,34,35,36,37,38]. However, there has recently been a trend of researchers reorienting themselves towards understanding the possible mechanisms of COVID-19 infection [39,40,41]. To our knowledge, few studies have targeted the evaluation of patients with asymptomatic/mild forms of the disease.

In our study, in asymptomatic/mild patients, the mean seroconversion time was day 12 for IgM and day 14 for IgG. In the first week, fewer than 40% of patients developed antibodies; the percentage increased beginning with day 15. Sun et al. (2020) showed that in most non-ICU patients IgM reached the peak in the second week after symptom onset [42]. Additionally, they observed that within one week after the symptom onset, the seropositive rates of IgM in non-ICU patients were 41.7% [42]. This is in line with our findings. There are some reported cases in which anti-SARS-CoV-2 antibodies were not detected at all, or at least they did not develop in the time frame proposed for the study [6,7,8,41]. This statement is supported by our results, too. In our study, we observed that IgM became detectable in most of patients (77%) with asymptomatic/mild forms beginning with day 7 of illness, while IgG became detectable at the end of the second week of illness in 95% of the tested patients. The week of illness with the highest mean IgG concentration was approximately the same as the week of seroconversion in other studies. We calculated the peak of the mean concentration for IgG as being in the fourth week. 

In 22.6% of patients with asymptomatic/mild forms, both immunoglobulins were absent in the tested sera. This can be explained in two ways: either they did not develop specific antibodies or they were not present during the study period. Twelve (38.7%) patients had a negative IgM test result in their first week of illness but had a positive test for IgG in the second or in the third week. This may be due to an earlier onset of disease than was declared. Eight (25.8%) patients developed IgM in the first 10 days, but they did not develop IgG until day 21, possibly due to a later IgG seroconversion.

Patients with moderate forms of COVID-19 developed IgM 5 days earlier than the asymptomatic/mild patients. The immune response in their sera was observed during the first week of the infection for IgM, and in the second week for IgG. The maximum mean concentration per week was reached in the first 7 days for IgM, and in the fourth week for IgG. This can be explained by an earlier onset of the suggestive symptoms of disease compared with the patients with the asymptomatic/mild form. Seventeen (45.9%) patients developed both IgM and IgG within the study period. A few patients (8.1%) were positive either only for IgM or only for IgG by the 14th day of hospitalization. The presence of IgM, but not IgG, within two weeks of hospitalization may be explained by IgM decline and IgG tardive production. We recorded six (13.6%) patients with moderate COVID-19 with undetectable antibodies by day 20. 

Only for some severe/critical patients were IgM detected in the first week of illness and IgG in the second week. The concentration of IgM reached its maximum value within 7 days, while the IgG concentration increased until the third week of the disease. One third of the patients developed both types of antibodies before day 14. Twelve (37.5%) patients were negative for IgM in the second week, but they tested positive for IgG at that point. On the other hand, six (13%) patients tested positive for IgM in the second week, but were negative for IgG until the third week. The results for the severe/critical patients are similar to those of other authors. Guo et al. (2020) [1] and Theel et al. (2020) [4] concluded that IgM can be detected during the first week after the onset of symptoms but IgG can be detected only at around 14 days. Wang et al. (2020) [2] observed that the average time of IgM occurrence was about 5 days, while for IgG it was about 14 days. Many other authors observed that IgM occurred in the serum of patients approximately 5–7 days after the onset of the disease, while IgG occurred after 10–21 days and could persist for a long time [6,7,8,41].

The life span of the anti-SARS-CoV-2 antibodies is uncertain. However, using the existing information on the other coronaviruses, we can assume that the anti-SARS-CoV-2 antibodies decrease over time (12–52 weeks after the onset of symptoms) [9]. Numerous SARS-CoV-2 reinfections have also been documented [9]. It is known that 90% of patients are positive for IgG two years after SARS-CoV infection, and in 50% of cases, these antibodies persist for over 3 years [19]. Zeng et al. (2020) stated that, in 80% of cases, IgM persisted until day 49 [6]. We observed an increase in IgG concentration until a peak in the fourth week. The concentration increase is important in the first 4 weeks, while the decrease is slow after the fourth week. Sun et al. (2020) [42] observed that the antibody response gradually increased for weeks 1–3 after the onset of the disease and that IgM reached a peak in the second week, while IgG antibodies continued to increase in the third week. Our results are similar to those obtained by Sun et al. (2020) [42] for all forms of the disease.

The absence of detectable antibodies could be due to the low sensitivity of the method (74.5% in first 7 days of disease). This may explain the variability in the antibody detection described so far by several authors due to the sensitivity of detection kits and the moment of appearance of the antibodies and their low titer [4,5,6,7,8].

Serological tests seem to be gaining popularity due to their low price, short time to results, use of common equipment, and accessibility of detection methods. The evolution of the infection can be predicted by measuring: IL-6, leucocyte count, neutrophil count, % neutrophils, NLR, PLR, CRP, urea, and creatinine, which tend to increase with COVID-19 severity, or lymphocyte count, % lymphocytes, eosinophil count, % eosinophils, hemoglobin, and eGFR, which decrease in correlation with the severity of the disease. 

Most of the asymptomatic/mild-form patients had elevated IL-6 concentrations. Hambali et al. (2020) also described a non-severe COVID-19 patient with persistently high IL-6 level [43]. Our patients with moderate, severe, and critical forms of disease had higher concentrations of IL-6. Zhang et al. (2020) [12] observed similar differences in IL-6 concentrations according to the form of the infection. We also detected certain blood parameters in the asymptomatic/mild form which were significantly increased, such as CRP, or were significantly lower, such as the eosinophil count and % eosinophils. Gu et al. (2021) mentioned that the WBC differential count may be abnormal in asymptomatic/mild COVID-19 patients [44].

IL-6 and CRP were significantly increased in the moderate form, while eosinophil count and eGFR were significantly lowered (Table 3, Table 4, Table 5, Table 6 and Table 7). IL-6, CRP, NLR, PLR, glucose, AST, urea, creatinine, and eGFR were significantly higher in our severe/critical COVID-19 patients. On the other hand, eosinophil count is significantly decreased in the same group (Table 3, Table 4, Table 5, Table 6 and Table 7). LOS did not correlate with % neutrophils, lymphocyte count, % lymphocytes, hemoglobin, or platelet count nor with the PLR. It is influenced by the creatinine level (moderate, positive correlation) and eGFR (moderate, negative correlation). IL-6, neutrophil count, % neutrophils, NLR, PLR, CRP, AST, and urea increased with the severity of the infection. The lymphocyte count, % lymphocytes, eosinophil count, % eosinophils, and hemoglobin decreased with the severity of COVID-19 (Table 3, Table 4, Table 5, Table 6 and Table 7). As the IL-6 concentration increased, the CRP, % neutrophils, NLR, and PLR increased and the lymphocyte count, % lymphocytes, hemoglobin, and eGFR decreased. The NLR and PLR rose as CRP and AST increased. NLR and PLR were also positively strongly correlated one with each other, meaning that both increased at the same time.

The statistical comparison tests showed a significant difference between males and females only in IgG secretion and NLR: males develop a higher immune response and a higher NLR value compared to women. Boon et al. (2021) stated that females develop a weaker immune response after infection and vaccination due to estrogen secretion [45].

We used the one-way ANOVA test to compare the blood parameters according to the severity of COVID-19. According to the f test of the One-Way ANOVA, there were significant differences between the three severity groups of COVID-19 in IL-6, CRP, % neutrophils, lymphocyte count, % lymphocytes, eosinophil count, % eosinophils, NLR, PLR, and urea (Table 7). On the other hand, concerning the leucocyte count, neutrophil count, hemoglobin, platelet count, LOS, glucose, ALT, AST, creatinine, and eGFR, there were no significant differences between the three groups. The test of homogeneity of variances showed that the variances of the IL-6 were not homogeneous. Based on the results, in terms of IL-6, there were significant differences between patients with asymptomatic/mild disease, those with moderate disease, and those with severe/critical disease, respectively, meaning that patients in the asymptomatic/mild category had a significantly lower level of IL-6 compared with those with the moderate or severe/critical form of disease. The statistical analysis of CRP, NLR, and PLR showed a significant difference between the severe/critical and the asymptomatic/mild or moderate forms, respectively. Since the variances of the % neutrophils and % lymphocytes were homogeneous, we applied Tukey’s post hoc test. The results allowed us to conclude that there were significant differences in % neutrophils and % lymphocytes between the severe/critical forms, the moderate forms, and the asymptomatic/mild forms of COVID-19. The variances of the lymphocyte count were not homogeneous. Upon applying the Games–Howell test, we found that there was a significant difference between the asymptomatic/mild forms of the disease and the moderate and severe/critical forms, respectively, regarding the lymphocyte count category. For the result of the homogeneity of variances test for % eosinophils, we used Tukey’s post hoc test. This test showed a significant difference between patients with asymptomatic/mild forms of disease compared to those with moderate and severe/critical forms, respectively. 

Our results are similar to those of Hou et al. (2020) [46]. They concluded that severe COVID-19 disease was associated with significantly increased neutrophils, infection biomarkers (such as CRP), and cytokine levels and decreased lymphocyte counts [46]. Xu et al. (2020) showed that the IL-6 concentration was significantly higher in fatal forms of COVID-19 when compared with patients with mild cases [47,48].

Ding et al. (2021) compared the blood parameters of the patients with mild/moderate cases to patients with severe COVID-19 [49]. We noticed that no significant differences in WBC counts and hemoglobin were observed between the compared groups of patients. The WBC differential count in the severe/life-threatening cases exhibited an increase in neutrophil count and a decrease in lymphocyte and eosinophil counts. NLR and CRP levels increased more in the severe COVID-19 group compared with the mild/moderate group [49].

The review by Velavan and Meyer (2020) [13] showed that the WBC was within the reference interval in all the tested cases. Mo et al. (2021) concluded that platelets count was normal or slightly lower in severe cases [50]. Some authors found that the neutrophil count [51,52], creatinine [53], and glucose [54] were significantly increased in critically ill patients. Other authors found that lymphocyte count [55,56,57] and eosinophil count [52] declined in most cases. The CRP value was described as increasing in most cases [50,57]. Chen et al. [53] observed that the IL-6 concentrations increased according to the severity of the disease from the mild to the critical form (mild ˂ severe ˂ critical). Our results are in line with the results described above. 

Our findings are also similar to those of Hachim et al. (2020), who proved that raised urea concentration and decreased lymphocyte count could predict the admission to an intensive care unit [14]. Man et al. (2021) argued that NLR and PLR have been proven to be reliable markers in COVID-19 patients and are increasingly correlated with CRP [15]. Similar findings were found in our study.

Xia et al. (2021) [58] and Xiang et al. (2021) [59] established the presence a high proportion of early kidney function injury in COVID-19 patients at admission. The decline of eGFR and the escalation of creatinine on admission were related to poor prognosis. Our results support their conclusions. The absence of an increase in LOS in critically ill patients and the lack of correlation between LOS and this form of the disease is due to the fact that, in several critically ill patients, death occurred after just 14 days.

In asymptomatic/mild-form patients, the IL-6 and CRP concentrations were significantly higher and eosinophil count was significantly lower compared with the reference interval. In the moderate form, the concentration of IL-6, CRP, and IgG were significantly higher compared with the reference interval, while eosinophil count and eGFR were significantly lower. In severe/critical COVID-19 patients, IL-6, CRP, NLR, PLR, glucose, AST, urea, creatinine, and eGFR were significantly higher compared with the reference interval, while eosinophil count was significantly lower. 

As IL-6, CRP, NLR, PLR, lymphocyte count, and eosinophil count are strongly correlated with the form of the disease (Table 3), we can state that they may represent a minimum set of markers to be tested as the best predictors for the aggressive evolution of COVID-19. Additionally, as ROC analyses revealed, lymphocyte count, lymphocyte percentage, eosinophil count, and eosinophil percentage can predict severe forms of COVID-19.

Comorbidities are correlated with the severe form of the disease and IL-6 concentration. Patients who had more comorbidities were associated with a higher concentration of IL-6 and developed a more severe form of the disease. Additionally, patients who had diabetes, malignancies, or renal dysfunction had more severe forms compared to the patients with cardiovascular diseases, mostly presenting critical forms that culminated in death. Our results are in line with Sanyaolu et al. (2020) who concluded that COVID-19 patients with history of hypertension, diabetes, and cardiovascular disease had bad prognoses. Additionally, chronic kidney disease patients and cancer patients are not only at risk for contracting the virus, but there is a significantly increased risk of death among these groups of patients [60]. The most frequent comorbidity encountered in our patients was hypertension (58.75% patients). Our results are similar to those of Franki et al. (2020), who observed that one of the leading comorbidities among COVID-19 deaths in NY, USA, was hypertension, as seen in 55.4% of their patients [61]. A large group of patients (40%) had two or more of the following comorbidities: cardiovascular, metabolic, renal, or endocrinological conditions. 

### 4.1. Strengths of the Study

A strong point of our study was the analysis of a statistically significant number of serum samples from patients with different forms of the disease, including asymptomatic patients, thus enabling us to obtain an image of how the body reacts when triggering the necessary physical response depending on each form of COVID-19. Most of the patients had mild or moderate forms of COVID-19. Another strength of the present study was that all the patients included in our cohort were infected at the beginning of the pandemic and all had a prompt hospital admission, so all the serum samples were collected at the onset of the disease, leading to the most correct detection as possible of the onset of the antibody response. In general, most authors have focused on searching for the risk factors or to understand possible mechanisms in severe or critical COVID-19; however, this paper also explored the variations of the blood parameters, not only in moderate or severe COVID-19 cases but also in mild or asymptomatic patients.

Our study explored a multitude of factors that may influence the severity of the disease (e.g., sex, age, comorbidities) and correlated a wide range of hematological, inflammatory, biochemical, and serological tests. Although the study was carried in a single hospital, patients from all northeastern Romania were hospitalized there and were included in the study.

### 4.2. Limitations of the Study

Our study has several shortcomings. We were not able to assess the antibody titers at longer time intervals for all patients due to their different LOS. We could not follow the evolution of the patients after their hospital discharge due to the lack of availability of all patients; only a small number of patients, statistically insignificant, returned for clinical–biological reevaluation at the hospital.

## 5. Conclusions

The amplitude and the time-point of the appearance of a detectable immune response depended on the severity of COVID-19 and the sensitivity of the test that was used—seroconversion being more frequently reported in severe cases. IL-6 was increase in all forms of COVID-19, with a major rise in severe and critical patients. IL-6, neutrophil count, % neutrophils, NLR, PLR, CRP, AST, and urea rose with the increased severity of the SARS-CoV-2 infection, and lymphocyte count, % lymphocytes, eosinophil count, % eosinophils, hemoglobin decreased with the increased severity of COVID-19. These changes allow us to conclude that all COVID-19 patients must be surveilled carefully—patients who develop asymptomatic or mild forms of disease should not be neglected.

The negative evolution of COVID-19 can be presumed by measuring the IL-6, CRP, lymphocytes, and NLR, which are strongly correlated. The lymphocyte count and the eosinophil count can predict the severe form of COVID-19. Patients with associated comorbidities developed more severe forms of COVID-19. The LOS depended on the kidney injury in COVID-19 patients. 

## Figures and Tables

**Table 1:** Mean value of blood parameters by severity of the disease.

Severity of Disease/ Blood Parameter	Unit	Mild/ Asymptomatic	Moderate	Severe/ Critical	Reference Interval
IL-6	pg/mL	19.57	73.26	149.36	0–7
Anti-SARS-CoV-2 IgG	AU/mL	25.82	36.81	64.17	<1
Leucocyte count	* 10^3^/µL	6.09	6.54	8.39	4–10
Neutrophil count	* 10^3^/µL	3.64	4.66	6.77	2–8
% neutrophils		57.49	64.85	74.59	45–80
Lymphocyte count	* 10^3^/µL	1.82	1.29	1.01	1–4
% lymphocytes		30.79	25.28	16.09	20–45
Eosinophil count	* 10^3^/µL	0.09	0.05	0.03	0–0.5
% eosinophils		1.78	0.80	0.48	0–5
Hemoglobin	g/dL	13.27	12.51	11.99	11.7–17.3
Platelet count	* 10^3^/µL	247.41	221.59	234.53	150–380
NLR		2.32	4.01	8.26	
PLR		153.60	185.72	257.77	
CRP	mg/L	19.83	41.96	116.67	0–5
Glucose	mg/dL	143.06	115.66	142.32	70–115
ALT	UI/L	78.47	46.21	46.94	5–40
AST	UI/L	45.71	38.83	61	5–37
Urea	mg/dL	37.94	45.52	75.18	15–50
Creatinine	mg/dL	1.25	1.48	2.28	0.5–0.9
eGFR	mL/min/1.73 m^2^	77.68	76.42	61.57	90–120
LOS	days	22.76	27.34	24.77	

Abbreviations: IL-6 = interleukin 6; IgG = immunoglobulin G; NLR = neutrophils/lymphocytes ratio; PLR = platelets/lymphocytes ratio; CRP = C reactive protein; ALT = alanine transaminase; AST = aspartate aminotransferase; eGFR = estimated glomerular filtration rate; LOS = length of stay. "*" is the symbol for the mathematical multiplication sign.

**Table 2:** The mean concentration, median, standard deviation, variance, and range of concentrations of the tested markers obtained in the present study.

Blood Parameters	Unit	Mean	Median	Standard Deviation	Variance	Range
IL-6	**pg/mL**	**80.70**	**15.04**	**145.72**	21234.75	499.50
WBC count	* 10^3^/µL	7.23	5.69	4.96	24.64	33.20
Neutrophils count	* 10^3^/µL	5.34	3.66	4.84	23.44	30.19
% neutrophils		67.43	66.75	15.49	239.93	62.30
Lymphocytes count	* 10^3^/µL	1.28	1.23	0.61	0.37	3.21
% lymphocytes		22.54	20.75	12.12	147.01	44.90
Eosinophils count	* 10^3^/µL	0.05	0.01	0.08	0.01	0.39
% eosinophils		0.87	0.25	1.39	1.93	7.00
Hemoglobin	g/dL	12.45	12.70	2.01	4.05	9.30
Platelets count	* 10^3^/µL	232.58	214.50	94.94	9013.99	488.00
NLR		5.46	3.22	6.17	38.12	29.50
PLR		209.52	182.73	102.50	10507.04	469.16
LOS		25.33	23.00	8.96	80.28	41.00
CRP	mg/L	69.67	32.45	83.62	6993.11	365.78
Glucose	mg/dL	132.81	122.50	55.75	3108.43	411.00
ALT	UI/L	53.38	32.00	79.82	6371.25	548.00
AST	UI/L	49.71	33.00	50.53	2553.50	308.00
Urea	mg/dL	56.51	37.00	53.96	2911.87	314.00
Creatinine	mg/dL	1.77	0.95	2.14	4.60	10.50
eGFR	mL/min/1.73 m^2^	70.30	77.72	33.00	1089.32	138.35

Abbreviations: IL-6 = interleukin 6; NLR = neutrophils/lymphocytes ratio; PLR = platelets/lymphocytes ratio; CRP = C reactive protein; ALT = alanine transaminase; AST = aspartate aminotransferase; eGFR = estimated glomerular filtration rate; LOS = length of stay. "*" is the symbol for the mathematical multiplication sign.

**Table 3:** The *p* and *r* values of the correlations of serum parameters with the form of the disease.

Serum Parameters	*p*	*r*
IL-6	<0.001	0.388
IgG	0.043	0.227
neutrophil count	0.005	0.312
% neutrophils	<0.001	0.454
NLR	<0.001	0.491
PLR	<0.001	0.380
CRP	<0.001	0.496
AST	0.012	0.281
urea	0.026	0.248
lymphocyte count	<0.001	−0.453
% lymphocytes	<0.001	−0.504
eosinophil count	<0.001	−0.400
% eosinophils	<0.001	−0.422
hemoglobin	0.018	−0.265

*p* = statistical significance coefficient, *r* = correlation coefficient. Abbreviations: IL-6 = interleukin 6; IgG = immunoglobulin G; NLR = neutrophils/lymphocytes ratio; PLR = platelets/lymphocytes ratio; CRP = C reactive protein; AST = aspartate aminotransferase.

**Table 4:** The *p* and *r* values of the correlations of serum parameters with the IL-6 concentration.

Serum Parameters	*p*	*r*
IgG	0.045	0.216
% neutrophils	0.011	0.281
NLR	0.002	0.334
PLR	0.001	0.350
CRP	<0.001	0.512
lymphocyte count	0.002	−0.340
% lymphocytes	0.002	−0.349
hemoglobin	0.003	−0.329
eGFR	0.038	−0.234

*p* = statistical significance coefficient; *r* = correlation coefficient. Abbreviations: IgG = immunoglobulin G; NLR = neutrophils/lymphocytes ratio; PLR = platelets/lymphocytes ratio; CRP = C reactive protein; eGFR = estimated glomerular filtration rate.

**Table 5:** The *p* and *r* values for the correlations of serum parameters with the IgG titer.

Blood Parameters	*p*	*r*
leucocyte count	0.002	0.340
neutrophil count	<0.001	0.389
% neutrophils	<0.001	0.389
NLR	<0.001	0.423
CRP	0.004	0.324
ALT	0.003	0.324
IL-6	0.045	0.216
PLR	0.028)	0.245
AST	0.008	0.296
% lymphocytes	<0.001	−0.441

*p* = statistical significance coefficient; *r* = correlation coefficient. Abbreviations: IL-6 = interleukin 6; NLR = neutrophils/lymphocytes ratio; PLR = platelets/lymphocytes ratio; CRP = C reactive protein; AST = aspartate aminotransferase; ALT = alanine transaminase.

**Table 6:** Comparison of the values of the serum parameters with the reference values according to the form of the disease.

Serum Parameters		*p*
Asymptomatic/mild form	IL-6	0.002
	CRP	0.016
	IgG	0.011
	eosinophil count	0.005
	% eosinophils	0.005
Moderate form	IL-6	0.009
	CRP	0.002
	IgG	<0.001
	eosinophil count	<0.001
	eGFR	0.026
Severe/critical form	IgG	<0.001
	IL-6	<0.001
	CRP	<0.001
	NLR	<0.001
	PLR	0.001
	Glucose	0.027
	AST	0.013
	urea	0.047
	Creatinine	0.006
	eGFR	<0.001
	eosinophil count	<0.001

*p* = statistical significance coefficient. Abbreviations: IL-6 = interleukin 6; IgG = immunoglobulin G; NLR = neutrophils/lymphocytes ratio; PLR = platelets/lymphocytes ratio; CRP = C reactive protein; AST = aspartate aminotransferase; eGFR = estimated glomerular filtration rate.

**Table 7:** Significant differences between the blood parameters for the three subgroups.

Serum Parameters	*p*
IL-6	0.021
CRP	<0.001
% neutrophils	<0.001
lymphocyte count	<0.001
% lymphocytes	<0.001
eosinophils	0.038
NLR	0.001
PLR	<0.001
urea	0.024

*p* = statistical significance coefficient Abbreviations: IL-6 = interleukin; NLR = neutrophils/lymphocytes ratio; PLR = platelets/lymphocytes ratio; CRP = C reactive protein.

**Table 8:** The *p* and r values of the correlations of comorbidities with the severe form of COVID-19 and the IL-6 concentration.

	Severe Form of COVID-19	IL-6 Concentration
**Comorbidities**	*p* = 0.014 *r* = 0.274	*p* = 0.019 *r* = 0.262

## Data Availability

The data that supports our study is available in Supplementary materials.

## Supplementary Materials

The following supporting information can be downloaded at: https://www.mdpi.com/article/10.3390/microorganisms11020319/s1, Table S1. Demographic data and comorbidities of the patients in the study group. Patients are grouped according to the severity of COVID-19, Figure S1: ROC curves generated for biomarkers that best predicted the severe forms of COVID-19.
