# Supplementary material for: The Immune, Inflammatory and Hematological Response in COVID-19 Patients, According to the Severity of the Disease
Source: Microorganisms. 2023 Jan 27;11(2):319. doi: 10.3390/microorganisms11020319 (PMC9967162; doi:10.3390/microorganisms11020319)
Supplement: Supplementary file 1 [file microorganisms-11-00319-s001.zip › microorganisms-2164376-supplementary.pdf]

Supplementary Materials

Table S1. Demographic data and comorbidities of the patients in the study group. Patients are grouped according to the severity of COVID-19

No	Age	Gender	Comorbidities
**Asymptomatic**			
1	47	F	-*
2	54	F	Type 2 diabetes, class 2 obesity, hypertension
3	48	F	-*
4	59	F	Type 2 diabetes, hyper-thyroiditis
5	87	F	Hypertension, chronic kidney disease
6	52	M	-*
7	54	F	Type 2 diabetes, hypertension, osteoporosis
**Mild**			
1	71	F	Hypertension, class 2 obesity, dyspepsia, chronic venous insufficiency
2	33	M	Vitiligo
3	68	F	Insulin-requiring type 2 diabetes, dyslipidemia, hyperuricemia, subtotal thyroidectomy
4	49	F	-*
5	50	M	Brain tumor, hemiplegia, aphasia, cachexia
6	65	F	Insulin-requiring type 2 diabetes, hypertension, class 2 obesity, hyperalgic diabetic polyneuropathy, Parkinson's disease
7	51	F	-*
8	19	M	-*
9	30	F	-*
10	42	M	-*
**Moderate**			
1	65	F	Hypertension, Class III NYHA chronic heart failure, chronic ischemic cardiopathy, Parkinson's disease, gastroesophageal reflux, vertigo syndrome, hepatic steatosis, cerebral atherosclerosis, anxiety
2	51	M	Chronic kidney disease, hemodialysis
3	47	M	Decompensated alcoholic liver cirrhosis, Chronic alcoholism, Chronic pancreatitis
4	43	F	Hypertension, angina pectoris
5	51	M	Sinus tachycardia, hypercholesterolemia, chronic cholecystitis
6	49	M	-*
7	55	M	Insulin-requiring type 2 diabetes, hypertension, class 1 obesity
8	46	F	Class II obesity, hypo-thyroiditis associated with nodular goiter
9	53	M	-*
10	44	F	Allergic asthma

11	39	F	Hypertension, ventricular extrasystoles, morbid obesity, gastroesophageal reflux
12	32	F	Autoimmune thyroiditis
13	42	F	-*
14	81	F	Hypertension, hyperthyroidism
15	64	M	Hypertension, prostate benign hyperplasia
16	63	F	Hypertension, chronic kidney disease, hemodialysis
17	71	F	Type 2 diabetes, chronic coronary syndrome, operated gastric neoplasm, ileostomy
18	88	F	Hypertension
19	62	F	Allergic asthma, autoimmune thyroiditis
20	45	F	-*
21	51	M	Type 2 diabetes, chronic coronary syndrome, operated gastric neoplasm, ileostomy
22	48	M	Hypertension, class 1 obesity
23	38	F	-*
24	58	M	Hypertension
25	65	M	Type 2 diabetes, old personal pathological cardiovascular antecedents of acute articular rheumatism (severe aortic regurgitation, reduced atrial flutter), class II NYHA chronic heart failure, medium pulmonary hypertension, stage II chronic kidney disease
26	49	M	-*
27	48	F	Congenital atrial septal defect
28	51	F	Hypo-thyroiditis
29	32	M	-*
**Severe**			
1	84	M	Hypertension, ischemic cardiopathy
2	66	M	Hypertension, gout
3	64	M	-*
4	45	F	Class I obesity
5	58	F	-*
6	80	M	Hypertension, ischemic heart disease, heart failure, permanent electric cardio-stimulation, stent, complete intermittent atrioventricular block, aortic atheromatosis, hyper-cholesterolemia, prostate benign hyperplasia
7	61	M	-*
8	55	F	Hypertension, cardiomegaly, morbid obesity, hypo-thyroiditis, chronic venous insufficiency
9	67	F	Hypertension
10		M	-*
11	49	F	Autoimmune thyroiditis, psoriasis
12	56	M	Type 2 diabetes, hypertension, atrial fibrillation, sequels of cerebral vascular accident, acute renal failure associated to acute tubular necrosis, temporary hemodialysis, class I obesity

13	60	M	Hypertension, angina, chronic kidney disease, hemodialysis, Insulin-requiring type 2 diabetes associated with hyponatremia, diabetic nephropathy, diabetic retinopathy
14	46	F	Hypertension, toxic liver cirrhosis, chronic liver failure, ascites, portal hypertension
15	45	M	Type 2 diabetes, hypertension, left ventricular failure, chronic obstructive bronchitis, right nephrectomy, chronic renal failure
16	50	F	Spastic tetra paresis, stroke sequela, multiple sclerosis, cerebral atrophy, cerebellous syndrome, anxiety
17	65	F	Hypertension, chronic hepatitis C, class I obesity
18	66	F	Multiple cardiovascular antecedents
19	40	F	Class I obesity
20	74	F	Chronic kidney disease, hemodialysis, insulin-requiring type 2 diabetes (unbalanced), secondary arterial hypertension, intermittent atrio-ventricular block Mobitz 2 degree, permanent electric cardio stimulation, chronic ischemic cardiopathy, mitral insufficiency
21	56	F	Chronic kidney disease, chronic glomerular nephropathy, secondary arterial hypertension, right hemiplegia, aphasia, vascular epilepsy
22	47	M	-*
**Critical**			
1	53	M	-*
2	60	F	Hypertension, hypertensive cardiopathy, hypo-thyroiditis
3	42	M	Multiple cardiovascular and metabolic diseases
4	60	M	Class III obesity
5	70	M	Chronic kidney disease, aortic atheromatosis, multicentric hepatocarcinoma, hemorrhagic ascites
6	51	F	Hypertension, atrial fibrillation, chronic kidney disease, hemodialysis, class III obesity
7	77	F	Multiple cardiovascular and metabolic diseases
8	68	M	Insulin-requiring type 2 diabetes (unbalanced), gastric cancer
9	46	M	Multiple cardiovascular and metabolic diseases
10	45	F	Multiple cardiovascular and metabolic diseases
11	68	M	Toxic hepatic cirrhosis, hepato-renal failure
12	64	F	Chronic kidney disease, hemodialysis, secondary arterial hypertension, hypo-thyroidia, liver vasculitis, chronic ischemic cardiopathy, metabolic cardiomyopathy

* No comorbidities

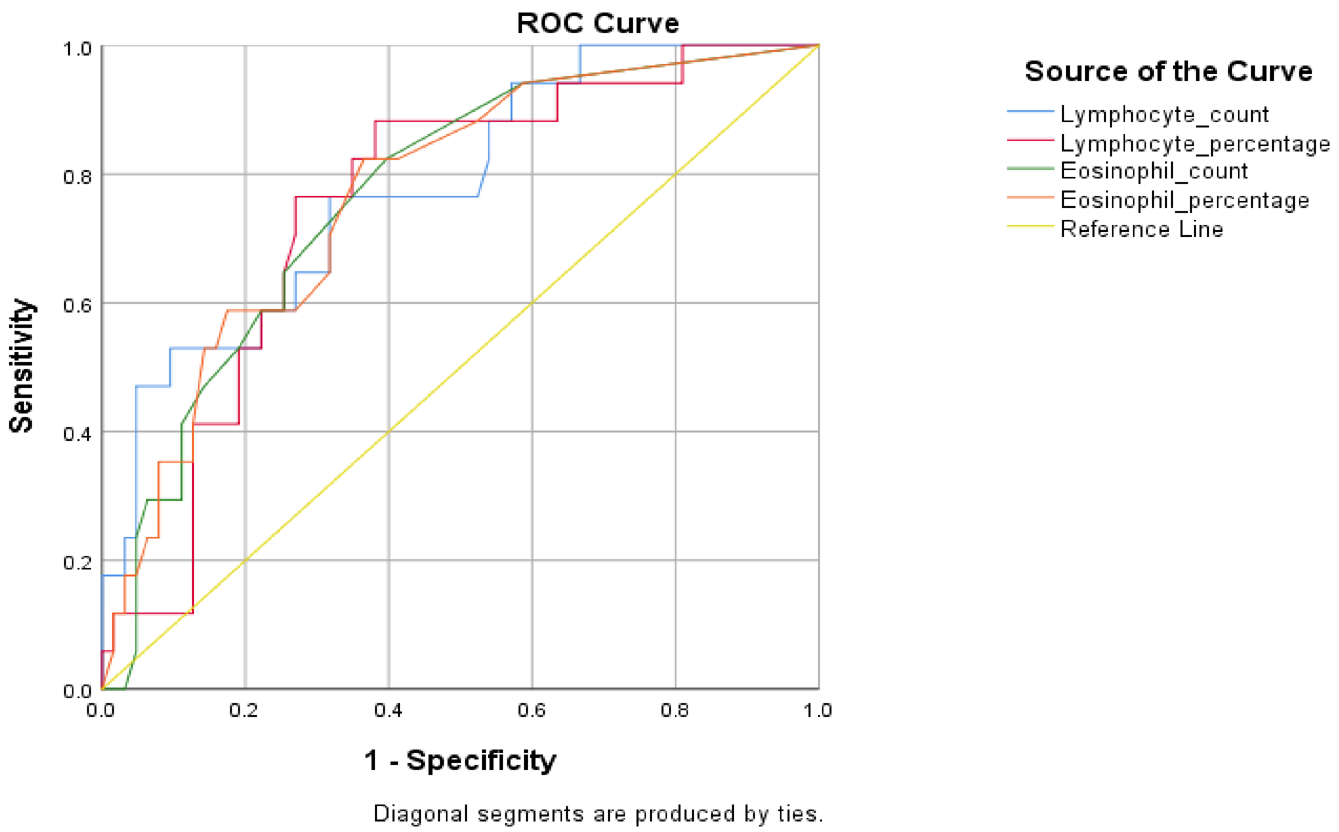

Figure S1: ROC curves generated for biomarkers that best predicted the severe forms of COVID-19. *Lymphocyte count, lymphocyte percentage, eosinophil count and eosinophil percentage equally predicted the severe forms of the infection in our study group. For this analysis, the larger values of the test variables indicate stronger evidence of a severe COVID-19 form, while the AUC values between 0.7-0.8 define a good capacity of discrimination.*
